# Establishment of a highly efficient conjugation protocol for *Streptomyces kanamyceticus* ATCC12853

**DOI:** 10.1002/mbo3.747

**Published:** 2018-11-17

**Authors:** Shuman Zhang, Tiansheng Chen, Jia Jia, Liwen Guo, Huizheng Zhang, Chao Li, Renzhong Qiao

**Affiliations:** ^1^ State Key Laboratory of Chemical Resource Engineering Beijing University of Chemical Technology Beijing China; ^2^ State Key Laboratory of Natural and Biomimetic Drugs School of Pharmaceutical Sciences Peking University Health Science Center Beijing China

**Keywords:** conjugal transfer, gene knockout, kanamycin, kanamycin B, *Streptomyces kanamyceticus*

## Abstract

Kanamycin B as the secondary metabolite of wild‐type *Streptomyces kanamyceticus *(*S. kanamyceticus*) ATCC12853 is often used for the synthesis of dibekacin and arbekacin. To construct the strain has the ability for kanamycin B production; the pSET152 derivatives from *Escherichia coli* ET12567 were introduced to *S. kanamyceticus* by intergeneric conjugal transfer. In this study, we established a reliable genetic manipulation system for *S. kanamyceticus*. The key factors of conjugal transfer were evaluated, including donor‐to‐recipient ratio, heat‐shock, and the overlaying time of antibiotics. When spores were used as recipient, the optimal conjugation frequency was up to 6.7 × 10^−6^. And mycelia were used as an alternative recipient for conjugation instead of spores; the most suitable donor‐to‐recipient ratio is 1:1 (10^7^:10^7^). After incubated for only 10–12 hr and overlaid with antibiotics subsequently, the conjugation frequency can reach to 6.2 × 10^−5^ which is sufficient for gene knockout and other genetic operation. Based on the optimized conjugal transfer condition, *kanJ* was knocked out successfully. The kanamycin B yield of *kanJ*‐disruption strain can reach to 543.18 ± 42 mg/L while the kanamycin B yield of wild‐type strain was only 46.57 ± 12 mg/L. The current work helps improve the content of kanamycin B in the fermentation broth of *S. kanamyceticus *effectively to ensure the supply for the synthesis of several critical semisynthetic antibiotics.

## 1 INTRODUCTION

Kanamycin, one of the most important subclasses of aminoglycoside antibiotics, has been widely used to treat serious infections caused by gram‐positive and gram‐negative bacteria by interfering in protein synthesis (Robati, Arab, Ramezani, Langroodi, & Abnous, 2016). The semisynthetic derivatives of kanamycin, such as amikacin and dibekacin. Dibekacin and arbekacin are widely used to solve the issue of resistance effectively (Deguchi, Yokota, Koguchi, & Nakane, 1990; Watanabe, Goi, Hara, & Sugano, 1987). Our group has successfully designed and optimized the synthesis processes of dibekacin and arbekacin from kanamycin B (KB) with good yields (Qiao, Zhang, Zhou, & Li, 2017). KB is generally extracted from the broth of *Streptomyces kanamyceticus*. However, KB is not the main product, the yield of KB is only 5%–10% (Ni et al., 2011). The inadequate supply of KB seriously confines the output and leads to the high price of these crucial antibiotics. To solve the problem of KB in short supply, it is urgent and significant to construct engineered strain with the high‐yield ability of KB.

Many efforts have been made to make *S. kanamyceticus* produce more KB relatively. Thapa, Oh, Lee, and Liou (2007) reported the heterologous expression of the kanamycin biosynthetic gene cluster (pSKC2) in *Streptomyces venezuelae* YJ003, but the isolated compound from the transformant was verified to be KA rather than KB. Ni, Li, Yang, and Huang (2011) demonstrated that genes *aprD3*, *aprQ*, and *aprD4* were disrupted together in *Streptomyces tenebrarius* H6, which blocked apramycin and oxyapramycin biosynthesis and increased the production of carbamoyl KB. Subsequently, the disruption of *tacA* made the mutants produce KB instead of carbamoyl KB. The work demonstrated that the subtle genetic engineering could significantly improve the expected production and eliminate the undesired one. In our study, we hope to change the distribution of the fermentation products in *S. kanamyceticus* and make KB become the main product. Studies of kanamycin biosynthetic pathways provide information and direction for strain improvement. Park's team discovered that there are two parallel pathways to synthesize KA and KB, in which the first glycosyltransferase *kanF* accepts both UDP‐Glc and UDP‐GlcNAc as cosubstrates but preferentially transfers the former (Nepal, Oh, & Sohng, 2009; Park, Park, Nepal, & Han, 2011). However, in 2012, Sucipto reported that *kanJ* and *kanK* are responsible for the transformation from KB to KA, that is, KB would be the direct biosynthetic precursors of KA (Sucipto, Kudo, & Eguchi, 2012). These findings lay the solid foundation for the construction of high KB‐producing mutants from wild‐type *S. kanamyceticus*.

In this study, we knocked out *kanJ* and found that the knockout of *kanJ* led to the accumulation of KB. The KB yield of the *kanJ*‐disruption strain was 10.7‐fold higher than that of the original strain. Recently, Gao reported a similar research and got similar conclusions (Gao, Wu, Sun, Ni, & Xia, 2017). Compared with Gao's work, we established a reliable genetic manipulation system for *S. kanamyceticus* by optimization of conjugation conditions including donor‐to‐recipient ratio, heat‐shock, and overlaying time of antibiotics. Mycelia are used as recipient rather than traditional spores and there are no same reports in *S. kanamyceticus*. In our experiments, we found that the conjugation frequency of mycelium was higher than that of spores. The highest conjugation frequency of mycelium was 7.9‐fold higher than that of spores.

## 2 MATERIALS AND METHODS

### 2.1 Microorganisms, plasmids, media, and culture conditions

The microorganisms and plasmids used in this study are listed in Table 1. The Mannitol‐Soy‐agar (MS) medium and seed medium were used for conjugal transfer and selection of *S. kanamyceticus *mutant. Genomic DNA was isolated from wild‐type *S. kanamyceticus*, which was also used as the host. Both *S. kanamyceticus* and its mutants were cultivated in 250‐ml shaken flasks, with seed medium contained 15 g soluble starch, 4.0 g yeast extract, 0.5 g K_2_HPO_4_, and 0.5 g MgSO_4_ in 1.0 L tap water. After incubation at 28°C for 30 hr, the fermentation medium was inoculated with 3 ml (10% [vol/vol]) seed culture and incubated for 7 days. The fermentation culture medium contained 35 g soyal bean, 30 g maltose, 25 g soluble starch, 8 g Sodium nitrate, and 0.1 g ZnSO_4_ in 1.0 L tap water. Appropriate antibiotics were added to the media when needed at the following concentrations: apramycin, 35–50 μg/ml; nalidixic acid, 50 μg/ml; kanamycin, 25 μg/ml, and chloramphenicol, 25–50 μg/ml.

**Table 1:** List of plasmids and strains used in this work

Strains or plasmids	Description	Source or reference
*Streptomyces kanamyceticus*	Wild‐type, kanamycin producer	This laboratory
ΔkanJ mutant	Mutant of wild‐type *S. kanamyceticus* with a deletion of* kanJ*	This work
*Escherichia coli* DH5α	Cloning host	Hanahan (1983)
*E. coli* ET12567(pUZ8002)	Cloning host (methylation defective), used for conjugal transfer of DNA from *E. coli* to *Streptomyces *	Macneil, Gewain, Ruby, and Dezeny (1992)
pSA74	Vector with chloramphenicol resistant gene; Cm^R a^	This laboratory
pSET152	*Streptomyces* integration vector; Ap^R b ^	Flett, Mersinias, and Smith (1997)
pSQ202	pSET152‐derivative plasmid with a deletion of *ΦC31 *int	This work
pSQ202‐J	Recombinant plasmid for *kanJ* disruption	This work

Chloramphenicol resistant

Apramycin resistant

### 2.2 DNA manipulations

Isolation of the genomic and plasmid DNA, DNA ligations, and other DNA manipulations was performed according to the standard protocols. Table 2 summarizes the oligonucleotide primers, which were synthesized at Beijing Genomics Institute (Beijing, China). The polymerase chain reaction (PCR) enzymes were purchased from New England Biolabs (Beverly, MA, USA), while Fastdigest enzymes and T4 DNA ligase were from Thermo Fisher Scientific (Waltham, MA, USA). A Gel Extraction Kit (OMEGA Bio‐Tek, USA) was used to recover the target DNA fragments from agarose gels. All chemicals used in this work were molecular biology grade and commercially available.

**Table 2:** Primers used in this work

Gene	Sequence (5′−3′)	Restriction site
kanJ‐U F	CCC**AAGCTT**CGTGTACCAGCGGATGATGT	*Hin* *d*III
kanJ‐U R	C**GAGCTC**GTCCTGTTGGACGTGTCCTG	*Sac*I
Cmr F	C**GAGCTC**ACGTTGATCGGCACGTAAG	*Sac*I
Cmr R	GC**TCTAGA**TTAACGACCCTGCCCTGAAC	*Xba*I
kanJ‐D F	TGC**TCTAGA**CGATCGGCACACCCACGCG	*Xba*I
kanJ‐D R	CG**GAATTC**CTGGCGGATCAGCCACAGGG	*Eco* *R*I
kanJ F	TCTCGGCGATCCTTGCCGAGGGCATCGAG	
kanJ R	GGCGTACGGCGCGTACGCCTTCGGGC	

Note

**Bold**: Restriction sites

### 2.3 Construction of the plasmids

On the basis of pSET152, the plasmid pSQ202 was used to knockout the kanamycin biosynthetic gene (kanJ). A chloramphenicol resistance gene (Cmr) was required because no proper selection marker was available in pSQ202. First, the gene was amplified from pSA74 using the Cmr forward (F) and Cmr reverse (R) primers that had been inserted with *Sac*I and *Xba*I, respectively. Subsequently, to amplify a 1,020‐bp fragment containing the upstream sequence of *kanJ* (kanJ‐U) (GenBank ID: AJ628422.2), we used kanJ‐U F and kanJ‐U R primers, which had been inserted with *Hind*III and *Sac*I, respectively. To amplify a 1,073‐bp fragment containing the downstream sequence of *kanJ* (kanJ‐D) (GenBank ID: AJ628422.2), we used kanJ‐D F and kanJ‐D R primers, which had been inserted with *Xba*I and *EcoR*I, respectively. All three PCR fragments were digested with their corresponding enzymes and inserted to the *Hind*III/EcoRI cloning sites of pSQ202 generating plasmid pSQ202‐J.

### 2.4 Establishment and optimization of a conjugal transfer system

The intergeneric conjugation between *E. coli* and *S. kanamyceticus* was carried out as described previously by Mazodier, Petter, and Thompson (1989) with some modifications. The culture of the donor *E. coli *ET12567 (pSQ202, pUZ8002) containing conjugative plasmid was grown with the appropriate antibiotics to an optical density at 600 nm (OD600) of 0.4–0.6. To remove the antibiotics, the cells were collected and washed twice with Luria‐Bertani broth (LB) and then suspended in 1 ml LB. The *S. kanamyceticus* spores without heat treatment were washed twice and suspended in 2× YT broth at a concentration of 10^9^ per ml. Subsequently, the *S. kanamyceticus* spores were heated at 45–60°C for 10 min, incubated at 37°C for 2–3 hr and served as recipient. Donor and recipient cells were mixed and spread on MS plates and grown for 14–20 hr at 28°C. The effect of MgCl_2_ at 10–40 mM on conjugal efficiency was also evaluated. For conjugation with mycelia, *S. kanamyceticus* was incubated in seed medium for 48 hr at 28°C. Mycelia were collected and mixed with exponential donor cells, spread on the seed agar plates, and grown for 8–16 hr at 28°C. After incubation, the plates were covered with 1 ml water containing nalidixic acid (50 μg/ml) and apramycin (35 μg/ml) or chloramphenicol (50 μg/ml) as required and incubated at 28°C for 3–5 days until the exconjugants appeared.

The frequency of pSQ202 transfer was calculated based on the number of exconjugants on a selective plate divided by the number of recipient cells on a nonselective plate (Liu, Lin, Zhang, & Bian, 2007). The average frequency of three independent experiments was calculated.

### 2.5 Confirmation of the exconjugants by PCR

The exconjugants genomic DNA was extracted and then was confirmed by two PCR primers. The first pair of primers, Cmr F and Cmr R, was designed to prove that *Cmr* replaced *kanJ*; the expected PCR product was 898 bp. The second pair of primers, *kanJ* F and *kanJ* R, was used to verify whether the *kanJ* gene was present in the exconjugants. The amplification product was subjected to sequence analysis, and the result was compared with the sequence in GenBank.

### 2.6 Antibiotic isolation and analysis

After the wild‐type strain and *kanJ* mutant were cultured in fermentation medium at 28°C for 7 days, the culture broth was collected. Then, the pH of the culture broth was adjusted to 2 with H_2_SO_4_, and the acidified broth was stirred for 30 min and then centrifuged (1,680 g; 15 min). The supernatant was subsequently readjusted to pH 7 using NaOH and then re‐centrifuged (1,680 g; 15 min). The supernatant of the culture broth was prepared for bioassays and further separation and purification. Then further purification and product analysis were referred to a novel method that Qiao's laboratory has developed previously for the direct determination of KB in the presence of KA in fermentation broth using HPLC‐ELSD (Zhang, He, Zhang, & Liu, 2015). The isolated compound was also restored by dissolving the dried precipitates in water, and then, it was analyzed by electrospray ionization–mass spectrometry (ESI‐MS).

## 3 RESULTS

### 3.1 Effect of the concentration of MgCl_2_ on *E. coli* and *S. kanamyceticus* conjugation

**Figure 1:**
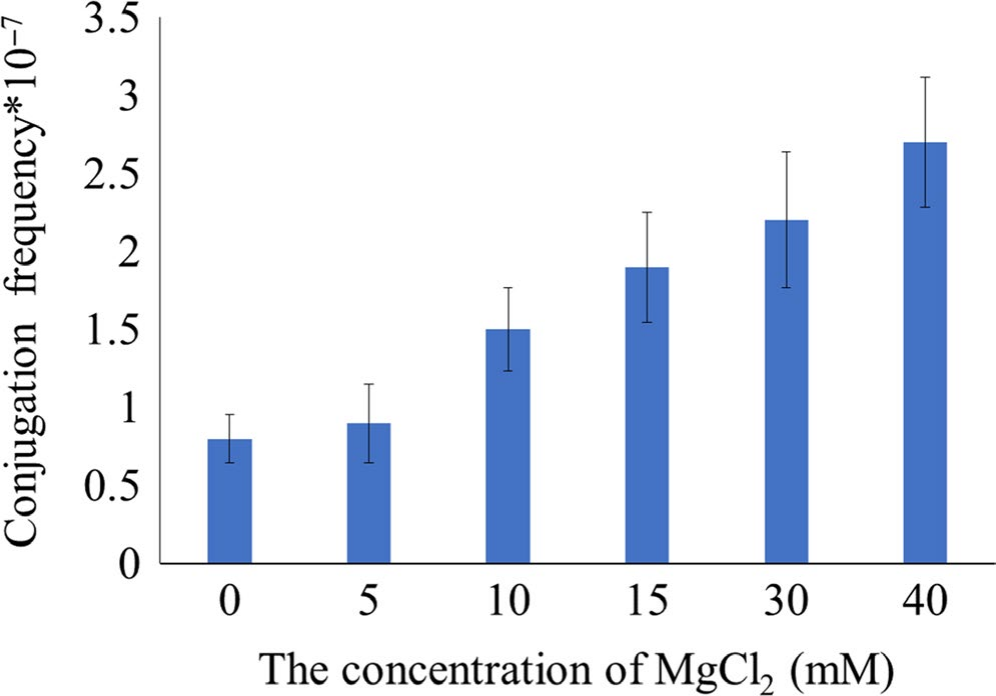
Effect of the concentration of MgCl_2_ on conjugation frequency with *Escherichia coli* ET12567 (pUZ8002/pSQ202) on MS agar medium

MgCl_2_ is commonly added to conjugation medium to improve the frequency of conjugation (Choi, Lee, Wang, & Kinoshita, 2004), and different *Streptomyces *strains correspond to respective optimal MgCl_2 _concentration. In our study, conjugation medium MS was further optimized with a supplement of 5, 10, 15, 30, 40 mM of MgCl_2_, respectively. The results revealed that the conjugation frequency enhanced with the increasing of MgCl_2_ concentration, as depicted in Figure 1. The most effective conjugation appeared in the medium containing 40 mM MgCl_2_. However, the formation and growth of spores were markedly inhibited when MgCl_2_ concentration was over 30 mM. The results suggested that 15 mM MgCl_2_ appeared to be the optimal concentration for conjugation of *S. kanamyceticus*.

### 3.2 Effect of heat‐shock on *E. coli* and *S. kanamyceticus* conjugation

The heat‐shock of the *S. kanamyceticus* spores was assessed in a temperature range between 45 to 60°C in order to determine the optimal temperature for conjugation. After heat‐shock, *Streptomyces* spores were precultured for 2–3 hr at 37°C to shorten the germination time. Different temperatures were selected to evaluate the effect of heat treatment on the conjugation frequency. As shown in Table 3, the rising temperature affiliated to the conjugation efficiency, and the maximum appeared at 55°C. However, the conjugation frequency rapidly decreased to 0.7 × 10^−7^ when the temperature was 60°C. Based on these results, 55°C was chosen as the best heat‐shock condition for the spores and used for all subsequent experiments.

**Table 3:** The effect of temperature of heat‐shock on *Escherichia coli* and *Streptomyces kanamyceticus* conjugation

*S. kanamyceticus*	Donor/recipient	Concentration of MgCl_2_ (mM)	Temperature of heat‐shock (°C)	Conjugation frequency
Spores	10^7^:10^8^	15	Nonheated	1.9 × 10^−^^7^
Spores	10^7^:10^8^	15	45	5.8 × 10^−^^7^
Spores	10^7^:10^8^	15	50	2.2 × 10^−^^6^
Spores	10^7^:10^8^	15	55	4.2 × 10^−^^6^
Spores	10^7^:10^8^	15	60	0.7 × 10^−^^7^

Note

Time of heat‐shock is 10 min.

### 3.3 Effect of donor‐to‐recipient ratio on *E. coli* and *S. kanamyceticus* conjugation

The ratio of donor‐to‐recipient cell number was a crucial parameter in the intergeneric conjugation of *Streptomyces (*Enriquez, Mendes, Anton, & Guerra, 2006). In order to establish an optimal spores recipient number for a given number of *E. coli *donor, we set up 4 series of matings. Different concentration of *S. kanamyceticus* spores and mycelia was mixed with *E. coli* ET12567 (pUZ8002, pSQ202) cells, respectively. The conjugation frequency was calculated and shown in Table 4. For spores recipient, no exconjugant was observed at the donor‐to‐recipient ratio of 10:1. The number of exconjugant colonies was found to increase with the number of spores recipient. The conjugation frequency increased to 6.7 × 10^−6^ when the ratio was up to 1:100. Subsequently, mycelia were used as recipient instead of spores. The highest conjugation frequency was obtained at the ratio of 1:1, which was 7.9 times higher than that optimized spores. However, few exconjugants were observed when the number of mycelia cells was 10^9^.

**Table 4:** The effect of donor‐to‐recipient ratio on *Escherichia coli *and *Streptomyces kanamyceticus* conjugation

*S. kanamyceticus*	Concentration of MgCl_2_ (mM)	Temperature of heat‐shock (°C)	Donor/recipient	Conjugation frequency	
Spores	15	55	10^7^:10^6^	—
Spores	15	55	10^7^:10^7^	2.0 × 10^−^^6^
Spores	15	55	10^7^:10^8^	4.2 × 10^−^^6^
Spores	15	55	10^7^:10^9^	6.7 × 10^−^^6^
Mycelia	0	—	10^7^:10^6^	3.2 × 10^−^^5^
Mycelia	0	—	10^7^:10^7^	5.3 × 10^−^^5^
Mycelia	0	—	10^7^:10^8^	1.3 × 10^−^^6^
Mycelia	0	—	10^7^:10^9^	2.6 × 10^−^^9^

Note

Time of heat‐shock is 10 min.

### 3.4 Effect of overlaying time of antibiotics on *E. coli* and *S. kanamyceticus* conjugation

Donor and recipient cells were mixed and spread on plates with incubation for 2–3 hr at 28°C. Subsequently, each plate was overlaid with 1 ml of antibiotic solution (1 ml of sterile water containing 50 μg of nalidixic acid against *E. coli *and 35 μg of chloramphenicol against *S. kanamyceticus*). The antibiotic overlaying time was another factor which may influence the conjugation frequency (Table 5). More exconjugants were obtained with the time prolonged from 16 to 18 hr. To some extent, premature overlaying weakened the growth ability of mycelia on the culture medium. Nevertheless, when the overlaying time of antibiotics was extended to 22 hr, the exconjugants of a single colony could not be observed on the plate and it induced false‐positive result of exconjugants. This suggested that the best overlaying time of antibiotics mix‐culture plates was 18–20 hr for using *S. kanamyceticus* spore as recipient, but only 10–12 hr for *S. kanamyceticus* mycelia.

**Table 5:** The effect of concentration and overlaying time of antibiotics on *Escherichia coli* and *Streptomyces kanamyceticus* conjugation

*S. kanamyceticus*	Concentration of MgCl_2_ (mM)	Donor/recipient	Temperature of heat‐shock (°C)	Overlaying time of antibiotics/hr	Conjugation frequency
Spores	15	10^7^:10^9^	55	14	2.2 × 10^−^^9^
Spores	15	10^7^:10^9^	55	16	2.9 × 10^−^^6^
Spores	15	10^7^:10^9^	55	18	6.1 × 10^−^^6^
Spores	15	10^7^:10^9^	55	20	6.7 × 10^−^^6^
Spores	15	10^7^:10^9^	55	22	a
Mycelia	0	10^7^:10^7^	—	8	1.2 × 10^−^^9^
Mycelia	0	10^7^:10^7^	—	10	5.3 × 10^−^^5^
Mycelia	0	10^7^:10^7^	—	12	6.2 × 10^−^^5^
Mycelia	0	10^7^:10^7^	—	14	a

a The antibiotics selection for exconjugants did not work, allowing the growth of both donor and recipient colonies. Time of heat‐shock is 10 min.

### 3.5 Construction of recombinant plasmids pSQ202‐J

**Figure 2:**
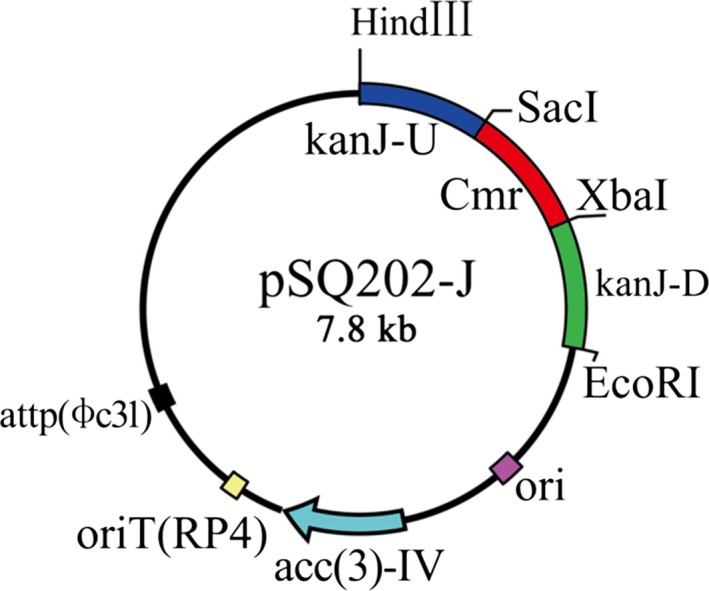
Construction and restriction map of plasmid pSQ202‐J

To construct the *kanJ* disruption plasmid, pSQ202 was used to delete the inner *Hind*III fragment (0.8 kb) of the ΦC31 int gene. The plasmid was constructed as described in materials and methods. The genetic organization and the restriction endonuclease map are shown in Figure 2.

### 3.6 Transformation of pSQ202‐J into *S. kanamyceticus*

**Figure 3:**
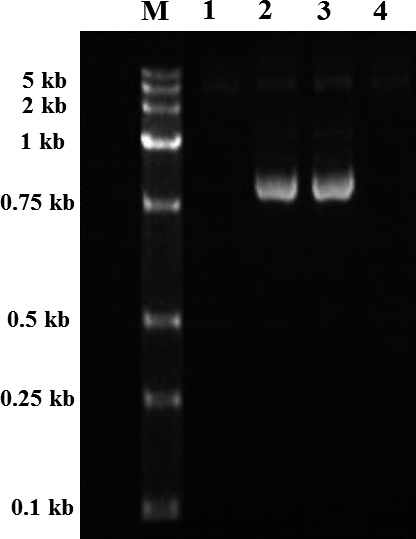
Verification of the recombination strains. PCR analysis with the genomic DNA from *Streptomyces kanamyceticus* and ΔkanJ mutant. Using primers Cmr F and Cmr R, PCR product from 1. wild‐type strain 2. ΔkanJ mutant; using primers kanJ F and kanJ R, PCR product from 3. wild‐type strain, 4. ΔkanJ mutant

According to the best conjugal transfer condition mentioned above, *E. coli* ET12567 (pUZ8002, pSQ202‐J) was used as donor to exconjugants with recipient of *S. kanamyceticus* mycelia. After the exconjugants appeared, single‐crossover exconjugants of *S. kanamyceticus* were obtained using a chloramphenicol as the selective marker. After three rounds of mycelia forming on the seed agar medium without antibiotic selection, chloramphenicol was added into the seed agar medium for double‐crossover exconjugants selection. The three double‐crossover exconjugants not sensitive to chloramphenicol were isolated to obtain ΔkanJ mutant. Then genomic DNA of exconjugants from ΔkanJ mutant was isolated and analyzed by PCR. As shown in Figure 3, ΔkanJ mutant showed an 898‐bp fragment with Cmr F and Cmr R as the primers and does not possess the 825‐bp *kanJ* sequence. The phenotypic characteristics including colony color, growth rates, and mycelium morphology were not altered in comparison with the original strain.

### 3.7 Effect of kanJ knockout in *S. kanamyceticus*

**Figure 4:**
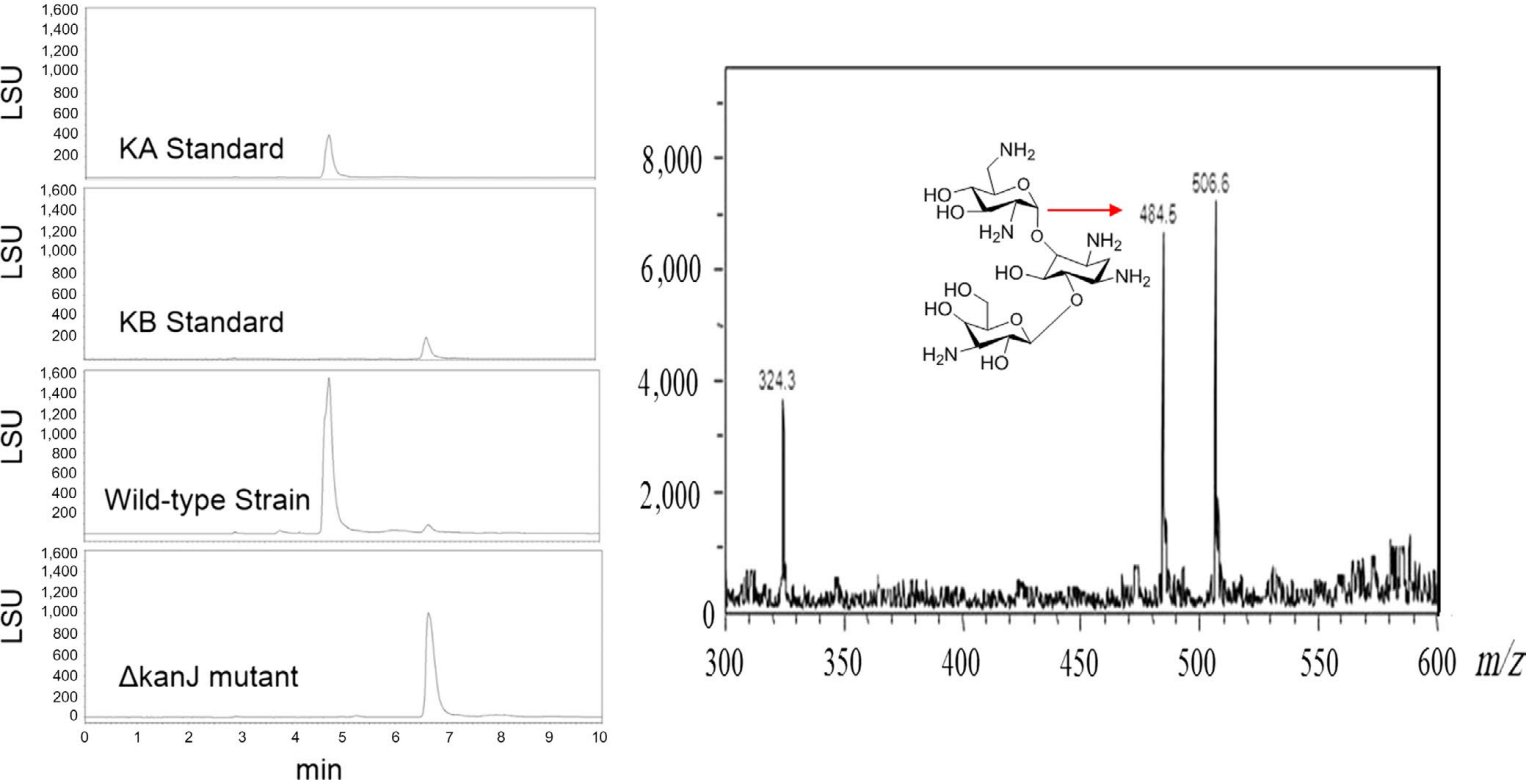
Metabolite analysis of wild‐type *Streptomyces kanamyceticus* and ΔkanJ mutant. (a) HPLC analysis of metabolites. (b) ESI‐MS analysis of the product of ΔkanJ mutant

Subsequently, the wild‐type strain *and *ΔkanJ mutant were fermented under the same conditions and their products were analyzed. HPLC‐ELSD analysis demonstrated that the products of wild‐type strain contained both KA and KB, while ΔkanJ mutant did not contain KA. And the main component of ΔkanJ mutant had the same retention time as KB (Figure 4a). The KB yield of wild‐type *S. kanamyceticus *was 46.57 ± 12 mg/L, and the KB yield of ΔkanJ mutant reached to 543.18 ± 42 mg/L. ESI‐MS demonstrated that the main component of the fermentation product had a molecular ion peak at 484.5 *m/z* (Figure 4b). These results confirmed that ΔkanJ mutant mainly produces KB.

## 4 DISCUSSION

There are different restriction‐modification systems in *Streptomyces* due to the diversity of them (Sadeghi, Soltani, Nekouei, & Jouzani, 2014). Transformation systems for several *Streptomyces* strains have been developed. Rocha (Rocha, Ruiz‐Villafán, & Manzo, 2018) reported a reliable genetic system for *Streptomyces peucetius *var.* caesius*, and the optimal conjugation frequency was up to 5 × 10^−4^. In Park, Jang, and Hwang (2012) work, a conjugal transfer system for *Streptomyces acidiscabies* was established. The highest conjugation frequency was 1.4 × 10^−3^. However, no conjugation efficiency of *S. Kanamyceticus* was reported to our knowledge. A rational genetic operating system of *S. kanamyceticus *should be established according to its own characteristics. In this work, a conjugal transfer system for the kanamycin producer *S. kanamyceticus *ATCC12853 was established and optimized successfully.

The results revealed that the donor‐to‐recipient ratio played a crucial role for affecting conjugation frequency in *S. kanamyceticus.* Different number of *E. coli* donor cells was used to explore the optimal one for a given number of spores. However, there were no obvious differences in the consequences and 10^7^ was applied for further experiments. As the recipient cells’ number increased, the number of exconjugant colonies increased simultaneously when spores were used in the tested range. For mycelia as recipient, in contrast, the increasing donor cells were not expected to achieve the improvement of conjugation frequency, which was not consistent with the results of spores.

The present work firstly demonstrated that *S. kanamyceticus* mycelia could be used quite effectively as recipient for intergeneric conjugation instead of spores. In addition, compared to 5–7 days culture time for spores, it was only 2–3 days when mycelia were used as recipient, and no heat‐shock operation was required. In particular, the optimal conjugation frequency achieved with *S. kanamyceticus* mycelia (6.2 × 10^−5^) was more satisfactory than that obtained with spores (6.7 × 10^−6^). It suggested that mycelia were more appropriate in *S. kanamyceticus* conjugation experiments.

Generally, several conjugal transfer conditions were screened, and the optimal one was confirmed to transfer the foreign plasmids into *S. kanamyceticus*. Kanamycin biosynthetic gene *(kanJ*) was knocked out through derivatives of pSET152, restraining the transformation of metabolites from KB to KA. The ferment result proved that we successfully made KB to be the main product in the fermentation broth. The highest KB yield of ΔkanJ mutant reached 585.33 mg/L, which was 10.7‐fold higher than that of the original strain. Thus, the current work provided a strategy to obtain adequate KB as raw materials for synthesis of dibekacin and arbekacin. Importantly, the possibility to create a new engineered strain, by using the similar genetic manipulation, is particularly significant for the future application of other antibiotics production.

## CONFLICT OF INTEREST

The authors declare that they have no competing interests.

## AUTHORS CONTRIBUTION

Shuman Zhang performed the experiment and edited the final version of the manuscript. Tiansheng Chen performed the experiment. Jia Jia, Liwen Guo, and Huizheng Zhang analyzed data. Chao Li and Renzhong Qiao contributed reagents and analytical tools. Shuman Zhang and Tiansheng Chen contributed equally.

## Data Availability

The data will be available on request from the corresponding authors.
